# Pharmacokinetic-pharmacodynamic modeling using combined time-kill data for meropenem and colistin or polymyxin B combination effects in *Acinetobacter baumannii*

**DOI:** 10.1128/aac.00274-26

**Published:** 2026-06-10

**Authors:** Hiie Soeorg, Sihong Lyu, Frank Kloprogge, Joseph F. Standing

**Affiliations:** 1 Great Ormond Street Institute of Child Health, University College London 4919, London, United Kingdom; 2 Institute for Global Health, University College London 4919, London, United Kingdom; 3 School of Pharmacy, University College London 4919, London, United Kingdom; 4 Department of Pharmacy, Great Ormond Street Hospital for Children 4956 , London, United Kingdom; Providence Portland Medical Center 25165, Portland, Oregon, USA

**Keywords:** model, pharmacodynamic, pharmacokinetic, colistin, polymyxin B, meropenem, *Acinetobacter baumannii*, combination

## Abstract

*Acinetobacter baumannii* is a high-priority pathogen with increasing antimicrobial resistance levels, requiring combination treatment. We aimed to develop a pharmacokinetic-pharmacodynamic model of meropenem with colistin/polymyxin B against *A. baumannii* based on a systematic review of time-kill experiments to inform the effectiveness of this combination. PubMed was systematically searched for all studies describing time-kill experiments of meropenem and colistin/polymyxin B combinations against A. *baumannii*. Time-kill data and strain metadata were extracted. A pharmacokinetic-pharmacodynamic model with logistic growth, sigmoidal Emax killing functions of monotherapies, time-dependent attenuation of drug effect, and general pharmacodynamic interaction for the combination effect was fitted. Simulations were performed to produce dose recommendations. A total of 21 eligible papers were found reporting data from 369 experiments on 53 strains. Minimum inhibitory concentration (MIC) on EC50 (concentration achieving half-maximum killing), inoculum on Emax (maximum killing rate), and beta-lactamases on Emax for meropenem were covariates and both drugs. EC50 of both drugs included interisolate variability. The model showed that colistin/polymyxin B increased meropenem Emax and decreased its EC50. No interisolate variability in the interaction was supported by the model. Simulations showed that in pneumonia, undetectable growth at 24 h can be achieved with a probability of >90% only in strains with meropenem MIC ≤16 mg/L. The model suggests that colistin/polymyxin B enhances meropenem activity across a diverse set of strains but does not achieve adequate target attainment in resistant strains (colistin/polymyxin B MIC ≥1 mg/L and/or meropenem MIC >8 mg/L). The presence of acquired beta-lactamases reduces the effect of meropenem.

## INTRODUCTION

*Acinetobacter baumannii* has become increasingly resistant to antimicrobial agents, such as carbapenems, which has risen among bloodstream infection-causing strains in Europe from 48.5% in 2017 to 70.8% in 2021 (1). This has driven widespread investigation and use of combination regimens, such as polymyxins with carbapenem (2).

Meta-analyses of *in vitro* time-kill studies have shown that meropenem as a combination agent for colistin and polymyxin B shows high synergy rates (60.4%–98.3%) (3, 4) and low antagonism rates (0%–1%) (3, 5). However, these meta-analyses have a disadvantage of pooling effects achieved with different concentrations while categorizing synergy as a binary variable (defined either by a fractional inhibitory concentration index (FICI) ≤0.5 (checkerboard/Etest) or by a ≥ 2 log10 colony-forming count (cfu)/mL reduction by the combination versus the most active monotherapy). This limits what can be concluded about the combination effect across clinically relevant exposures.

Semi-mechanistic pharmacokinetic-pharmacodynamic (PKPD) models could extrapolate beyond concentrations used in time-kill experiments, describe the variability of the extent of synergy (6), and identify possible subgroups with different synergistic effects. Mostly, the number of strains used in PKPD models is low, often one (7–9). This small number of isolates prevents accurate prediction of how combination regimens perform against strains of various characteristics, i.e., limiting generalizability of the findings to other *A. baumannii* strains (6, 8). Considering the multitude of *in vitro* time-kill experimental studies performed (26 reported for polymyxin-carbapenem combination in 2015 [4]), a large pooled data set could be generated for improved estimation of the combination effect. This could allow us to evaluate the combination effect in a stratified way, possibly shedding light on heterogeneity of the effect. This improved understanding could explain non-superiority in terms of mortality, clinical, or microbiological cure of this combination to colistin alone in a randomized clinical trial (OVERCOME) (10).

We aimed to develop a PKPD model of meropenem with colistin/polymyxin B against *A. baumannii,* combining available time-kill experiments from the research literature to quantify the extent and variability of the interaction of the drugs across a diverse set of strains.

## MATERIALS AND METHODS

### Systematic search

PubMed was systematically searched for all studies describing static time-kill or hollow-fiber infection experiments. The search was performed on 9 June 2025 using the term “acinetobacter baumannii AND (time kill OR time-kill OR hollow fiber OR hollow-fiber OR hollow fiber OR hollow-fiber)”. All retrieved papers were screened for the presence of cfu counts vs time data presented either in figures or tables, the antibiotics tested (alone or in combination), and the pathogen species studied. Papers were screened by two authors (SL and HS). Studies testing meropenem in combination with colistin or polymyxin B against *A. baumannii* were selected.

### Data extraction

Time-kill data were extracted manually using Online Plot Digitizer (https://plotdigitizer.com/). Sampling time points were recorded as those reported in the Materials and Methods section, unless absent. The limit of quantification (LOQ) of the sampling method in each paper was recorded, and points below the LOQ (BLOQ) were recorded as the LOQ and indicated to be BLOQ using a binary indicator. The strain identifier and its metadata (minimum inhibitory concentration [MIC], resistance elements) reported in the paper was recorded. In case of no reported MIC, authors were contacted before excluding the study. Studies reporting cfu/mL counts > 10^12^ were excluded due to biological implausibility of such values.

MIC breakpoints used for categorizing isolates as susceptible or resistant were based on EUCAST guidelines: meropenem, susceptible if MIC ≤2 and resistant if MIC >8; colistin, susceptible if MIC ≤2 and resistant if MIC >2 (11). In the analysis, acquired beta-lactamase was defined as the presence of meropenem-degrading beta-lactamases OXA-23, OXA-24/40, OXA-58, OXA-72, VIM-1, or NDM-1 (12, 13), excluding OXA-51 and OXA-69 as intrinsically present (14, 15) in *A. baumannii*.

### Modeling

NONMEM version 7.6 was used for fitting a pharmacokinetic-pharmacodynamic model. A stepwise approach to fitting the model was taken as follows: (i) the growth control data, (ii) meropenem or colistin/polymyxin B monotherapy data, and (iii) combination data. The population parameter values of fixed effects and variance of random effects estimated in each step were fixed and carried forward to the next step to stabilize parameter estimation. BLOQ observations were handled in the model using the M3 penalized likelihood approach (16).

The base model was a logistic growth model with sigmoidal Emax killing functions (with Emax showing maximum kill rate and EC50 potency) of monotherapies, time-dependent attenuation of drug effect, and Bliss independence with general pharmacodynamic interaction (GPDI) for the combination effect, similarly to Aubry *et al*. (17).

Bacterial growth was modeled as logistic growth with growth rate K_growth_, bacterial count (B), and carrying capacity parameter (B_max_), combined with killing rate K_kill_:


$$\frac{dB}{dt}=\left(K_{\text{growth}}\cdot \left(1-\frac{B}{10^{B_{\text{MAX}}}}\right)-K_{\text{kill}}\right)\cdot B$$

Logistic growth was used to reflect the biological limitation of bacterial expansion due to finite nutrients, space, and accumulation of metabolic waste in *in vitro* systems, resulting in a progressive slowing of growth at higher population densities. K_growth_ represents the net bacterial replication rate, including both growth and natural cell death, which cannot be reliably estimated as separate processes due to insufficient information content in the available *in vitro* data.

Initial bacterial burden was set from the inoculum parameter:


$$B\left(0\right)=10^{INOC}$$

Time-dependent attenuation of the effect of each drug was represented by a fraction of reduced killing effect. This component was intended to capture a reduction in effective antibacterial activity over time, potentially reflecting degradation of meropenem (18–20) and an increase in less susceptible bacterial subpopulations, including resistant and persister cells. For meropenem, switching from maximum killing (R_MERO,off_), which was the initial state, to the reduced killing (R_MERO,on_) occurred with attenuation rate K_atten,MERO_ during exposure to meropenem (meropenem concentration C_MERO_ > 0):


$$\frac{dR_{MERO,on}}{dt}=k_{atten,MERO}\cdot I\;\left(C_{MERO}>0\right)\cdot R_{MERO,off}\;$$

$$\frac{dR_{MERO,off}}{dt}=-k_{atten,MERO}\cdot I\left(C_{MERO}>0\right)\cdot R_{MERO,off}$$

Time-dependent attenuation of the effect of colistin/polymyxin B was modeled analogously:


$$\frac{dR_{COLPMB,on}}{dt}=k_{atten,COLPMB}\cdot I\left(C_{COLPMB}>0\right)\cdot R_{COLPMB,off}$$

$$\frac{dR_{COLPMB,off}}{dt}=-k_{atten,COLPMB}\cdot I\left(C_{COLPMB}>0\right)\cdot R_{COLPMB,off}$$

Attenuation of colistin/polymyxin B killing was intended to capture a reduction in effective antibacterial activity, potentially reflecting binding of colistin/polymyxin B to lipopolysaccharide (21) and enrichment of less susceptible bacterial subpopulations, including resistant and persister cells.

Drug killing effect was dependent on inoculum, continuously for meropenem and as reduced killing only if inoculum was 8 log10 cfu/mL in the case of colistin/polymyxin B. The inoculum effect potentially reflects a reduction in the amount of effective drug available per bacterial target in the presence of high bacterial load, resulting in lower target occupancy and therefore lower apparent killing (22). For meropenem, drug effect was also dependent on the presence of acquired beta-lactamases. Drug-drug interaction was modeled as a colistin/polymyxin B-dependent modification of meropenem maximal effect and potency. Interaction terms were described as saturable functions of colistin/polymyxin B concentration.


$$E_{max_{MERO,inoc}}=E_{max_{MERO}}\cdot {\left(\frac{INOC}{6}\right)}^{\gamma_{MERO}}$$

$$E_{max_{COLPMB,inoc}}=E_{max_{COLPMB}}\cdot {\left(\gamma_{COLPMB}\right)}^{I\left(INOC=8\right)}$$

$$K_{\text{MERO}}=\frac{E_{max_{MERO,inoc}}\theta_{BL}\left(1+{\text{Interaction}}_{Emax}\right)\left(1-R_{MERO,on}\right)C_{MERO}}{EC50_{MERO}MIC_{MERO}\left(1-{\text{Interaction}}_{EC50}\right)+C_{MERO}}$$

$$K_{\text{COLPMB}}=\frac{E_{max_{COLPMB,inoc}}\left(1-R_{COLPMB,on}\right)C_{COLPMB}}{EC50_{COLPMB}MIC_{COLPMB}+C_{COLPMB}}$$

$${\text{Interaction}}_{Emax}=\frac{\Delta_{Emax}C_{COLPMB}}{EC50_{INTER}MIC_{COLPMB}+C_{COLPMB}}{\text{, Interaction}}_{EC50}=\frac{\Delta_{EC50}C_{COLPMB}}{EC50_{INTER}MIC_{COLPMB}+C_{COLPMB}}$$

The killing rate by combination was computed as the sum of single-drug killing terms with an interaction component (scaled to the larger single-drug Emax):


$$E_{ref}=max\left(E_{max_{MERO,inoc}},E_{max_{COLPMB,inoc}}\right)$$

$$K_{kill}=K_{MERO}+K_{COLPMB}-\left(\frac{K_{MERO}}{E_{max_{MERO,inoc}}}\right)\cdot \left(\frac{K_{COLPMB}}{E_{max_{COLPMB,inoc}}}\right)\cdot E_{ref}$$

Inoculum was estimated with intended inoculum set as a typical value (possible values 4, 5, 6, 7, and 8 log10 cfu/mL). Exponential interisolate variability (IIV) was tested for the growth rate, carrying capacity, inoculum, Emax, EC50, and change in Emax and EC50 in the GPDI model.

EC50 was modeled as a·MIC^b^, where a and b were estimated or fixed to 1, whichever provided a better fit to the data. The presence of beta-lactamases was tested as a covariate for Emax or EC50, inoculum for Emax, and whether the drug was colistin or polymyxin B for EC50. The experiment type (static or dynamic) was tested as a covariate for growth rate, carrying capacity, and attenuation rate of killing. These parameters were selected because they are plausibly influenced by environmental conditions. Static systems are closed environments with finite nutrients, accumulation of metabolic waste, no clearance of released beta-lactamases, and no added antibiotics to compensate for degradation, whereas dynamic experiments provide continuous medium exchange, removal of enzymes and debris, and maintenance of the intended concentration-time profiles.

Models were selected based on objective function value (decrease by at least 3.84, corresponding to *P* < 0.05, required for choosing a more complex model among nested models), parameter precision, and numerical stability (successful minimization, reasonable standard errors). The model fit was checked using goodness-of-fit plots, prediction-corrected visual predictive check (pcVPC), and posterior predictive check of BLOQ fraction.

### Simulations

Simulations were performed to describe the effect of dosing regimen of colistin (5 mg/kg loading dose and 1.67 mg/kg maintenance dose every 8 h) alone and in combination with meropenem (1 g every 8 h) used in the OVERCOME trial, which did not show superiority of combination therapy over colistin monotherapy (10). Most patients had pneumonia (70%–71%) and were infected by *A. baumannii* (77%–78%), with a median colistin MIC of 1 mg/L and meropenem MIC in 90%–91% cases > 8 mg/L. PK profiles in plasma for a 67-year-old male patient weighing 60 kg and with serum creatinine of 1.5 mg/dL were predicted from colistin (23) and meropenem (24) pharmacokinetic models in the literature. For the lung pharmacokinetic profile, lung penetration was assumed to be 100% for unbound colistin (25) and 25% for unbound meropenem (26). The unbound fractions were assumed to be 44% (27) and 62.5% (28), respectively. Bacterial counts were simulated, and the probability of target attainment (PTA) of the target of no growth at 24 h above LOQ of 10 cfu/mL was calculated for a strain with colistin MIC of 1 mg/L, meropenem MIC of 16 mg/L, and with or without acquired beta-lactamase. A total of 1,000 stochastic simulations were performed with interisolate and residual variability. An inoculum of 6 log10 cfu/mL was used with random variability of a normal distribution with mean 0 and standard deviation of 0.25.

Additional stochastic simulations were performed with higher meropenem doses (2, 4, 6, and 8 g infused over 3 h q8h) and against different meropenem MICs (4 to 256 mg/L) and colistin MICs (0.5 and 1 mg/L) with a fixed inoculum of 6 log10 cfu/mL. Additional simulations were performed to evaluate meropenem continuous infusion at steady-state concentrations corresponding to 1×, 4×, and 8× MIC. To ensure clinical plausibility, only dosing regimens that result in the steady-state concentrations ≤44.5 mg/L were considered, corresponding to the reported threshold associated with an increased risk of meropenem-related toxicity (29). For comparison of the effectiveness of the regimens against different strains, the PTA was calculated for targets of no growth at 24 h above LOQ of 10 cfu/mL and bacterial count at 24 h ≥ 2 log10 lower than inoculum.

## RESULTS

The search retrieved a total of 427 papers. Of these, 240 papers presented individual time-kill experiment curves of antibiotics against *A. baumannii* (PRISMA flowchart shown in Fig. S1). A total of 26 papers reported time-kill experiment results of meropenem in combination with colistin or polymyxin B, of which 21 were eligible (Table S1). Of the papers included in this study, five papers had performed hollow-fiber infection model, 18 papers had performed static time-kill experiments, and three developed PKPD model (7–9).

The papers described a total of 369 experiments (70 [19.0%] growth control, 104 [28.2%] meropenem, 46 [12.5%] colistin, 37 [10.0%] polymyxin B, 53 [14.4%] meropenem and colistin, 59 [16.0%] meropenem and polymyxin B) against 53 strains (one used in three papers, five in two papers; strains used in multiple papers were used by the same research groups, except for ATCC 19,606, which was used by different groups; a total of 60 strain-paper instances hereafter designated as isolates). The number of strains used in a study ranged from 1 to 8, with a median of 2 (IQR 2–4).

In static time-kill experiments of monotherapy, the number of different meropenem concentrations used (in multiples of MICs) was a median of 2.5 (IQR 2–3; maximum 9), and colistin/polymyxin B was a median of 2 (IQR 2-3; maximum 9). In combination treatment time-kill experiments, the number of different regimens was a median of 2 (IQR 1-3; maximum 8). The most commonly used meropenem concentrations in combinations were 1 × MIC (in 10 papers), followed by 0.5 × MIC and 0.25 × MIC (both in six papers) (Fig. S1). The most commonly used colistin/polymyxin B concentrations in combinations were 0.5 × MIC (in seven papers), followed by 1 × MIC (in five papers) (Fig. S2).

**Fig 1:**
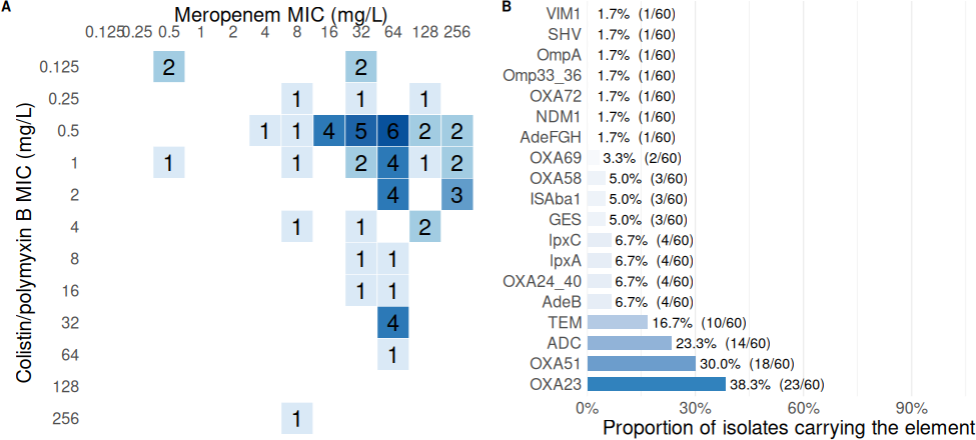
Characteristics of the isolates included in the model. (**A**) Joint distribution of minimum inhibitory concentrations (MICs) of meropenem and colistin/polymyxin B across isolates. Numbers indicate counts of isolates in each MIC combination. (**B**) The proportion of isolates reported to carry the respective resistance element (OXA-23, OXA-24/40, OXA-58, OXA-72, VIM-1, or NDM-1 defined as acquired beta-lactamases in subsequent analyses).

Of the overall 60 isolates, 57 were resistant to meropenem and 14 to colistin/polymyxin B (Fig. 1A), and 30 were reported to carry an acquired meropenem-degrading beta-lactamase (Fig. 1B).

In the growth control modeling step, an IIV term was retained only in carrying capacity (Table S2). Additional growth IIV terms improved objective function value but resulted in an unstable model (terminated, huge condition number, or implausible parameter precision). In the colistin/polymyxin B monotherapy model, an inoculum of 8 log10 cfu/mL decreased maximum killing rate, but there was no difference in the potency of colistin or polymyxin B (Table S2). In the meropenem monotherapy model, the presence of acquired beta-lactamases reduced maximum killing rate (Table S1). EC50 of both drugs included IIV. In the combination GPDI model, colistin/polymyxin B exposure increased meropenem Emax and decreased meropenem EC50. No IIV or effect of beta-lactamases on interaction was supported by the model (Table S2) due to non-convergence or high relative standard error, indicating an effect poorly informed by the data, respectively. The parameter estimates of the final model are shown in Table 1.

**TABLE 1:** Final pharmacokinetic-pharmacodynamic model colony-forming^*a*^

Parameter	Description	Estimate	RSE (%)
Growth			
K_growth_	Growth rate	0.33	30.0
B_max_	Carrying capacity, log10 cfu/mL	8.41	0.7
Interisolate variability, SD		0.11	10.6
Meropenem kill rate			
Emax_MERO_	Maximum killing rate at inoculum 6 log10, if no colistin/polymyxin B	3.47	12.7
K_atten,MERO_	Attenutation rate of meropenem killing	0.033	19.1
γ_MERO_	Power term for the inoculum effect on Emax_MER_	−0.63	24.0
$\theta_{BL}$	Change in Emax_MER_, if beta-lactamase present	0.83	9.2
EC50_MERO_	Fixed to MIC, if no colistin/polymyxin B		
Interisolate variability, SD		1.13	19.0
Colistin/polymyxin B kill rate			
Emax_COLPMB_	Maximum killing rate at inoculum < 8 log10	3.62	2.7
K_atten,COLPMB_	Attenuation rate of colistin/polymyxin B killing	0.075	6.0
$\gamma_{COLPMB}$	Change in Emax_COLPMB_, if log10 inoculum 8	0.005	11.7
EC50_COLPMB_	If MIC = 1 mg/L	0.33	13.2
Interisolate variability, SD		1.35	9.5
General pharmacodynamic interaction			
$\Delta_{Emax}$	Maximum increase in *K*_MER_ in the presence of colistin/polymyxin B	0.29	27.1
$\Delta_{EC50}$	Maximum decrease in *EC50*_MER_ in the presence of colistin/polymyxin	1 (fix)	
Residual variability		1.21	9.2
SD	Proportional residual error		

^*a*^ Final model equations are presented in the Supplementary text. MIC, minimum inhibitory concentration; RSE, relative asymptotic standard error; SD, standard deviation.

**Fig 2:**
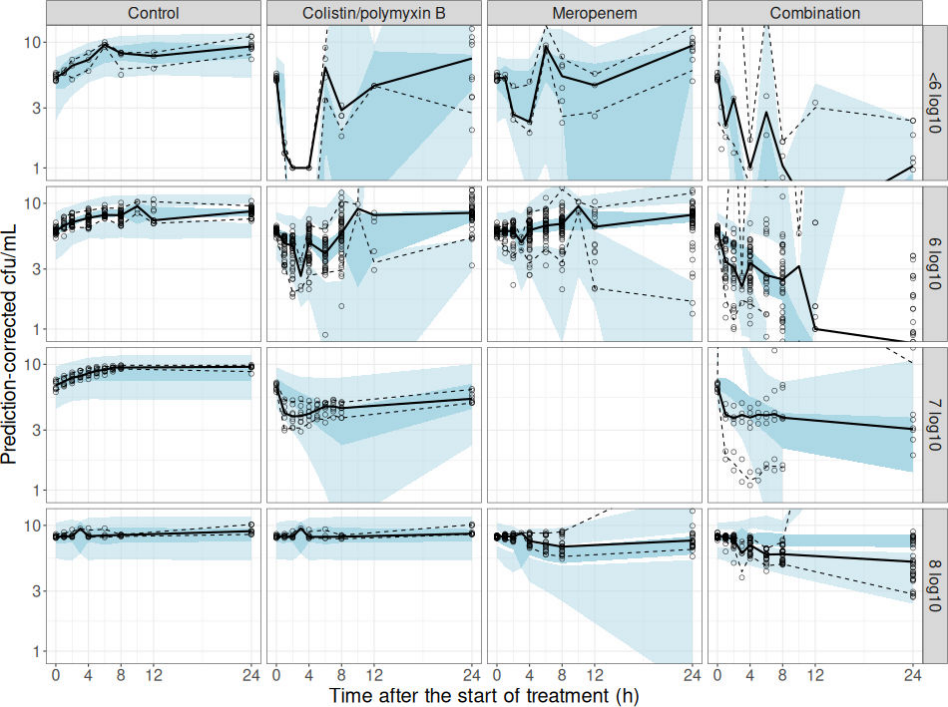
Prediction-corrected visual predictive check of the final pharmacokinetic-pharmacodynamic model, stratified by inoculum and exposure: no antibiotics (control), colistin/polymyxin B monotherapy, meropenem monotherapy, meropenem and colistin/polymyxin B combination. Dots show observations, solid lines median, and dashed lines show the 5th and 95th percentile of observations. Shaded areas show prediction intervals for the median, 5th, and 95th percentiles.

Goodness-of-fit plots showed no major systematic bias in predictions on the log10 cfu/mL scale (Fig. S3). The pcVPC stratified by inoculum and treatment reproduced the observed median trajectories and variability across inoculum and regimens (Fig. 2). Posterior predictive checks reproduced the fraction of BLOQ observations (Fig. S4).

**Fig 3:**
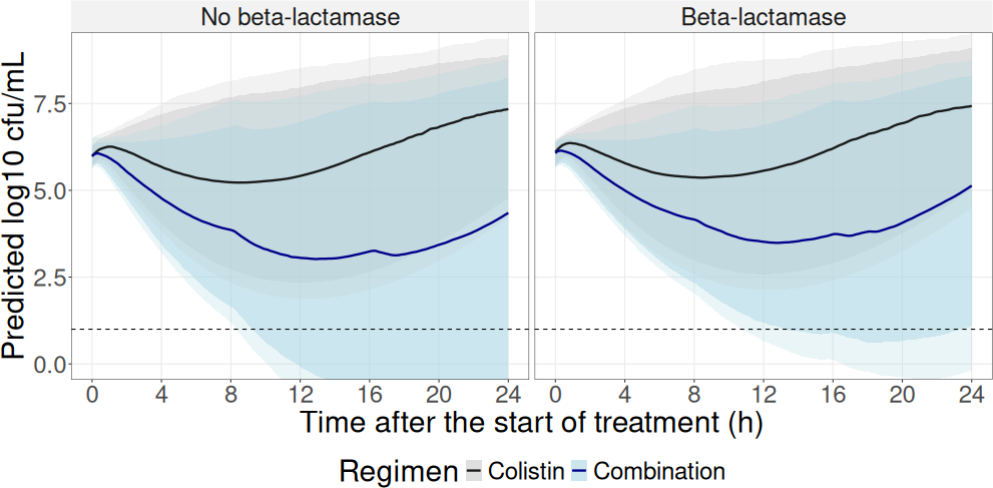
Simulated time-kill curves for colistin alone (gray) and in combination with meropenem (blue) against a strain with colistin MIC = 1 mg/L and meropenem MIC = 16 mg/L. Solid lines show the simulated median log10 cfu/mL trajectory, and shaded areas show the 90% (light shading; 5th–95th percentile) and 80% (dark shading; 10th–90th percentile) prediction intervals across 1,000 simulations. Panels are stratified by the presence/absence of acquired beta-lactamases. The dashed horizontal line indicates the lower limit of quantification (LOQ; 10 cfu/mL).

Both colistin monotherapy and combination therapy with meropenem as used in the OVERCOME trial had a high probability of growth above LOQ at 24 h (Fig. 3). Colistin monotherapy PTA of growth <LOQ at 24 h was 0%. In combination with meropenem, PTA was 22.7% and 9.3% for strains without or with acquired beta-lactamases, respectively.

**Fig 4:**
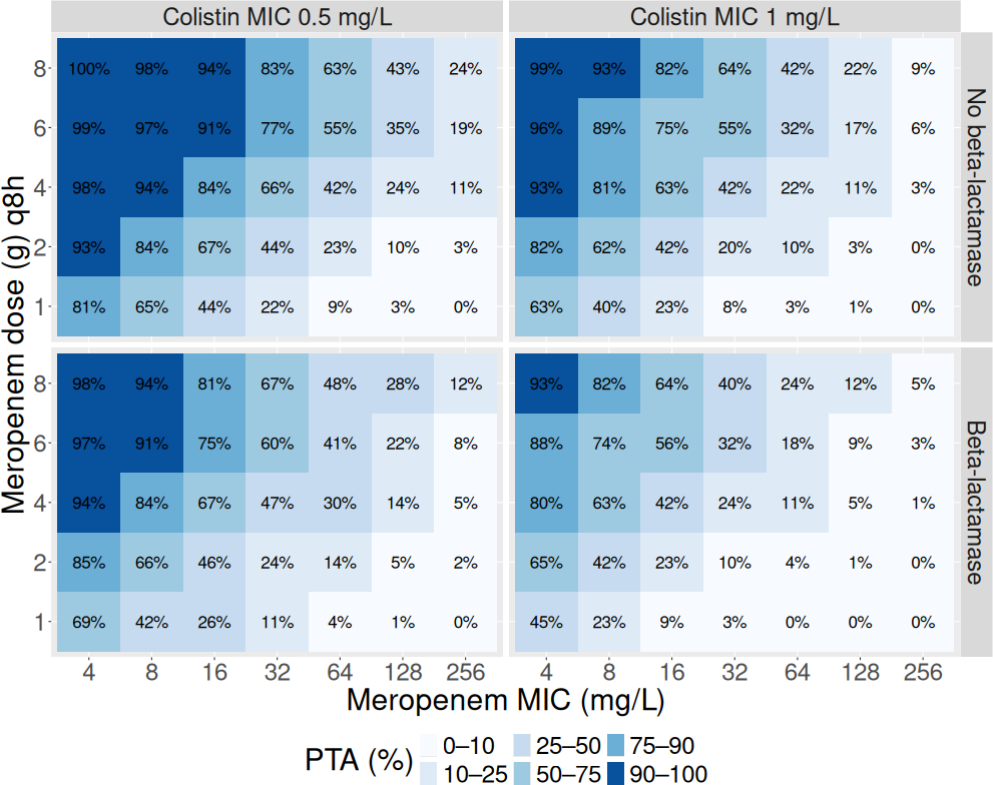
Probability of target attainment (PTA) stratified by minimum inhibitory concentration (MIC), the presence of acquired beta-lactamases, and meropenem dosing regimen. Tiles show whether PTA ≥0.9 was achieved at 24 h (target defined as bacterial count below the limit of quantification of 10 colony-forming units/mL) for each meropenem regimen in combination with colistin. Tile color indicates the extent of PTA (darker color shows higher PTA), and percentage shows PTA value.

**Fig 5:**
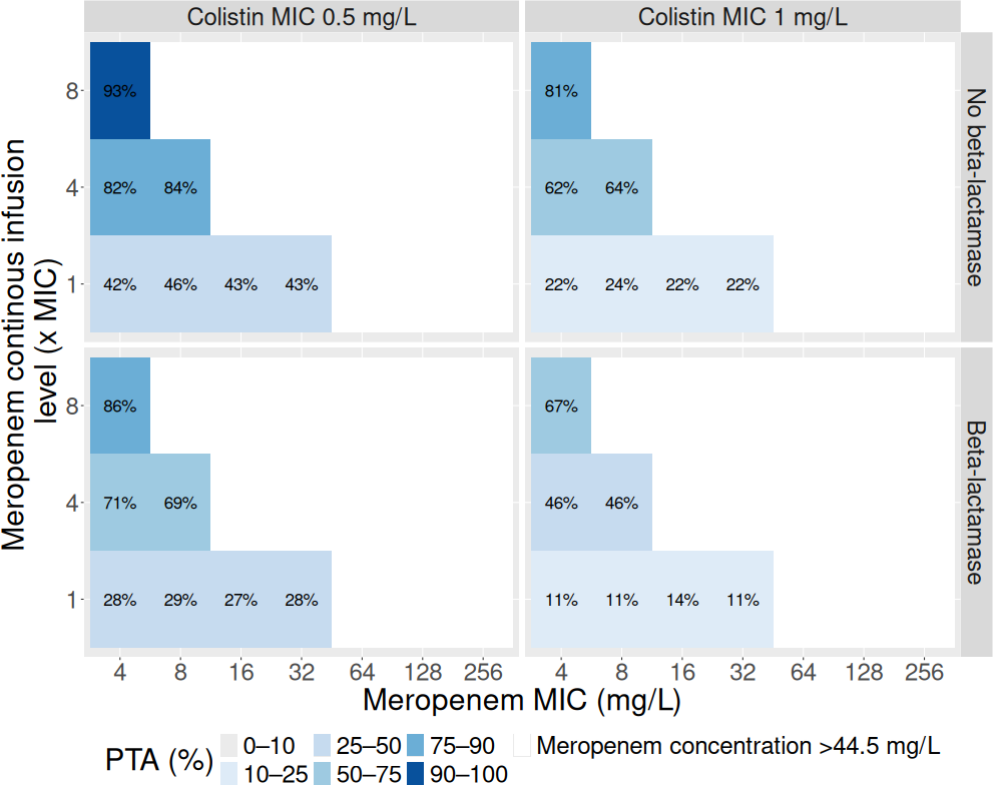
Probability of target attainment (PTA) for meropenem administered by continuous infusion achieving different levels of multiples of meropenem minimum inhibitory concentration (MIC) in combination with colistin, stratified by meropenem MIC, colistin MIC, and the presence of acquired beta-lactamases. Tiles indicate whether PTA ≥0.9 was achieved at 24 h (target defined as bacterial count below the limit of quantification of 10 colony-forming units/mL) for each steady-state meropenem concentration expressed as a multiple of the MIC. Tile color represents the extent of PTA (darker colors indicate higher PTA), and percentage shows PTA value. White tiles denote exposure combinations exceeding the predefined meropenem steady-state toxicity threshold (>44.5 mg/L) and were therefore considered infeasible.

The lowest meropenem dose was 2 g q8h infused over 3 h that achieved PTA ≥90%, but only against a strain with colistin MIC 0.5 mg/L, meropenem MIC of 4 mg/L, and without acquired beta-lactamases (Fig. 4). For strains with meropenem MIC >8 mg/L and colistin MIC of 1 mg/L, even a meropenem dose of 8 g q8h infused over 3 h did not reach PTA ≥90%. Continuous infusion achieved PTA ≥90% only in case of meropenem MIC of 4 mg/L and colistin MIC of 0.5 mg/L in the absence of acquired beta-lactamases and if the target was 8× MIC mg/L (Fig. 5). The PTA for the combination was substantially higher than that for meropenem monotherapy (Fig. S5).

Using a target of ≥2 log10 kill, PTA ≥90% was achieved against strains with meropenem MIC up to 8 mg/L, but only when colistin MIC was 0.5 mg/L and no acquired beta-lactamases were present (Fig. S6 and S7). Evaluated dosing regimens did not achieve PTA ≥90% for strains with meropenem MIC >8 mg/L or colistin MIC ≥1 mg/L.

## DISCUSSION

Pooled published meropenem and colistin/polymyxin B time-kill experiment data allowed the development of a semi-mechanistic PKPD model that identified the impact of acquired beta-lactamases on meropenem activity beyond what is explained by MIC. According to the model, meropenem activity is potentiated by colistin/polymyxin B, but the data did not support the estimation of variability in the extent of this interaction. Simulations of various dosing regimens showed beta-lactamase-associated differences in the probability of no growth at 24 h and that high meropenem doses are needed for growth suppression in the case of pulmonary infections.

Several features of the model are overall consistent with prior work. First, the inoculum effect has been shown for both meropenem and colistin/polymyxin B (30, 31) in *A. baumannii*. However, in the case of colistin/polymyxin B, we saw a threshold inoculum effect, where 8 log10 markedly attenuated killing in contrast to a continuous effect in previous literature (30). Second, we saw substantial variation of EC50 around MIC. Previous studies have shown that meropenem EC50 can be very similar despite 256-fold differences in MICs (9). Additional variability of EC50 around MIC can also arise from different MIC testing methods used, which may result in different MICs (32). In this data set, 15 of 21 studies used broth microdilution or stated adherence to CLSI guidelines, with the remaining studies not reporting the method or used various methods (Etest, VITEK, or disk diffusion). Due to the dominance of broth microdilution and diversity of other methods, assessing the impact of MIC testing methodology would not have been feasible, but interisolate variability could potentially compensate for the potential impact. Third, the typical colistin/polymyxin B EC50 value was lower than the MIC in our model. Indeed, colistin and polymyxin B achieve greater killing than meropenem at 1× MIC (33) and even at 0.5× MIC, initial killing can be substantial (34). Finally, our model did not support a potential difference between the effect of colistin and polymyxin B. Overall, the agreement between colistin and polymyxin B potency is very high, with >99% of MICs within a 1-fold difference (35) and similar synergy rates with various antibiotics (36). This is in line with inconclusive results from meta-analyses of *in vitro* time-kill studies, which have shown the colistin and meropenem combination exhibiting a lower synergy rate compared with polymyxin B (60.4% vs 98.3%) (3) or higher synergy rate (84.9% vs 63.4% [4]; effect size 0.87 vs 0.82 [37]).

The model allowed us to test the effect of acquired beta-lactamases on killing parameters and thereby evaluate their impact on the choice of dosing regimen. Despite scaling meropenem EC50 by MIC, the presence of acquired beta-lactamases was associated with an additional reduction in the maximal killing rate (Emax). One plausible explanation is that beta-lactamase activity decreases the effective meropenem concentration, producing an inoculum-effect-like attenuation of killing. Consistent with this, inoculum effects have been reported more frequently in beta-lactamase-producing bacteria (38). Thus, the beta-lactamase effect of reducing maximum killing rate in our model could partially be due to a higher effective enzyme burden, similar to the effect of higher inoculum. PKPD modeling of single strains also estimated that meropenem maximum killing rate is smaller if acquired beta-lactamase OXA-23 is present, despite similar EC50, though not always (8, 9). Our simulations showed that the presence of acquired beta-lactamases does affect the effectiveness of dosing regimens, warranting taking this bacterial characteristic beyond MIC into account as well. Commonly found beta-lactamases like OXA-23, OXA-24, OXA-58, VIM, and NDM (39) were present in our data set, supporting the relevance of this finding in a real-world setting.

The finding that colistin/polymyxin B augments meropenem killing activity is in line with high synergy rates reported in previous literature, supporting the potential of this combination therapy for treatment. Our model suggested an interaction effect of colistin/polymyxin B on both Emax and EC50. Considering the mechanisms of action of the drugs, this is plausible. First, colistin/polymyxin B permeates the membrane, allowing beta-lactams to enter the cell more easily (40) and thus reducing its EC50. Indeed, polymyxin B or colistin reducing meropenem EC50 has been shown before (8, 9). Second, the permeated outer membrane is destabilized and allows faster killing of bulged inner membrane cells (41), thus increasing Emax. Nevertheless, the doses needed to benefit from the synergy of meropenem and colistin/polymyxin B are high, possibly explaining the non-superiority of the combination over colistin monotherapy in a clinical trial (10).

Among the reasons for the non-superiority of the combination regimen in a clinical trial is the lack of synergistic effect in some strains (24.7%) (42). However, our model could not support between-strain variability in interaction parameters because including variability led to non-convergence and non-identifiability. There could be various reasons for that. First, the synergy rates reported from meta-analyses show very high rates and very few antagonistic strains. Thus, the lack of interisolate variability in the model could indeed indicate a robust and consistent interaction between the drugs. Notably, even in the case of polymyxin-resistant strains, synergy rates with carbapenems can be very high (79.8% vs 80.6% in the case of polymyxin-susceptible strains) (4). Between-strain variability could be better seen in other Gram-negative species like *Klebsiella pneumoniae* and *Pseudomonas aeruginosa*, in which case synergy rates are lower than in *A. baumannii* (effect size of meropenem and colistin combination was 0.12, 0.43, and 0.87, respectively (37); synergy rates of meropenem and polymyxins 34%, 24%, and 86% [5]). Second, heterogeneity of experimental conditions may have contributed to large variability, further complicating IIV estimation. For example, an incubation temperature of 37°C may result in more potent killing than 35°C (43). Additional conditions that can affect results include pH, nutritional composition of the broth (44), and laboratory materials (45). Temperature was not reported in four studies and broth was not reported in one study; however, 19 of 21 studies used (cation-adjusted) Mueller-Hinton broth. Given the extent of missing information and the dominance of a single broth type, these factors were considered insufficiently informative for formal covariate testing. Other experimental conditions were frequently not reported at all, further limiting the feasibility of systematically accounting for such sources of heterogeneity. Finally, because IIV was already incorporated in the meropenem and colistin/polymyxin B EC50 parameters, the ability to additionally estimate IIV on the interaction term may have been limited because only a limited number of concentrations, alone and in combination, were mostly tested per strain. This is the caveat of using pooled data that were not specifically designed for elucidating variability in interaction effect.

The non-superiority of the meropenem and colistin/polymyxin B combination in the OVERCOME trial (10) may reflect insufficient antibiotic exposure. Indeed, in our simulations, the regimen corresponding to the clinical trial showed a low probability of target attainment, suggesting that the meropenem dose used was not effective enough against resistant strains. According to our study, no dosing regimen achieved adequate PTA for strains with colistin MIC of 1 mg/L and meropenem MIC of 16 mg/L. Continuous infusion, which is generally preferred in critically ill patients to improve beta-lactam target attainment (46), also did not improve PTA, possibly because of the low penetration rate of meropenem into epithelial lining fluid. Even using a less stringent target of ≥2 log10 killing at 24 h did not improve PTA sufficiently to be adequate for strains with colistin MIC of 1 mg/L and meropenem MIC of 16 mg/L. Overall, this suggests that the combination is suboptimal for resistant strains (colistin MIC ≥1 mg/L and/or meropenem MIC >8 mg/L).

Several limitations of the study must be noted. First, the reporting of resistance mechanisms varied between the papers, with the possibility of not reporting some of them at all, exemplified by the lack of intrinsic OXA-51 reporting in some studies. This hampered us from building a model that takes resistance mechanisms into account, as recently termed a next-generation mechanism-based model (47). Second, we also did not account for possible degradation of meropenem, both non-enzymatic (18) and enzymatic due to beta-lactamases (19, 20), and binding of colistin/polymyxin B to materials (45) and released lipopolysaccharide (21). These processes are complex, depending on inoculum (20), beta-lactamase (19, 20), antibiotic concentration (20, 48, 49), materials (45), and experimental conditions (18), and are thus difficult to take into account without systematic experiments. However, because the data set included strains with diverse beta-lactamases, a range of inoculum sizes, and antibiotic concentrations, the model parameters describe the average effect of meropenem and colistin/polymyxin B across degradation and binding scenarios. Furthermore, model parameters may not be affected at all (49), or because carbapenemases effects may become evident only after 8 h (19), degradation may not affect killing parameters. Third, experimental conditions, like experiment volumes, between the individual studies may affect the precision and accuracy of the estimates.

### Conclusion

The developed global PKPD model suggests that colistin/polymyxin B enhances meropenem activity across a diverse set of strains. Although the combination effect is not strain-dependent, the presence of acquired beta-lactamase affects the activity of meropenem monotherapy, which in turn impacts the effectiveness of the combination regimen. The model demonstrates that the meropenem and colistin/polymyxin B combination is suboptimal against resistant strains with colistin MIC ≥1 mg/L and/or meropenem MIC >8 mg/L.

## Data Availability

All data used in the modeling and the final model code are publicly available on Zenodo (https://doi.org/10.5281/zenodo.19889677).

## SUPPLEMENTAL MATERIAL

Supplemental material aac.00274-26-s0001.docx Tables S1 and S2; Fig. S1 to S7.

ASM does not own the copyrights to Supplemental Material that may be linked to, or accessed through, an article. The authors have granted ASM a non-exclusive, world-wide license to publish the Supplemental Material files. Please contact the corresponding author directly for reuse.
