# Supplementary material for: Pharmacokinetic-pharmacodynamic modeling using combined time-kill data for meropenem and colistin or polymyxin B combination effects in Acinetobacter baumannii
Source: Antimicrob Agents Chemother. 2026 Jun 10;70(7):e00274-26. doi: 10.1128/aac.00274-26 (PMC13321832; doi:10.1128/aac.00274-26)
Supplement: Supplemental material — Tables S1 and S2; Fig. S1 to S7. [file aac.00274-26-s0001.docx]

**Supplementary material**

**Table S1.** Characteristics of published studies included in the pooled dataset

| Reference | Strains (n) | Experiments (n) | MIC measurement method | Broth | Temperature | Data points (n) | Meropenem static concentration (x MIC) | Meropenem dynamic regimens | Colistin/Polymyxin B static concentration (x MIC) | Colistin/polymyxin B dynamic regimens |
| --- | --- | --- | --- | --- | --- | --- | --- | --- | --- | --- |
| Abdul-Mutakabbir et al. (2021) | 3 | 12 | BMD | CAMHB | 37⁰ | 48 | 0–0.94 (n=4) |  | 0–0.5 (n=3) |  |
| Abdul-Mutakabbir et al. (2024) | 4 | 16 | BMD | MHB | 37⁰ | 64 | 0–0.5 (n=3) |  | 0–0.5 (n=5) |  |
| Bedenić et al. (2016) | 8 | 24 | BMD | NA | 37⁰ | 240 | 0–1.25 (n=4) |  | 0–4 (n=2) |  |
| Bian et al. (2019) | 4 | 34 | BMD | MHB | 35⁰ | 238 | 0–0.06 (n=3) | 1g over 3h q8h; 2g over 3h q8h; 0.5g over 0.5h q8h | 0–2 (n=5) |  |
| Ju et al. (2022) | 1 | 4 | BMD | CAMHB | 37⁰ | 24 | 0–1 (n=2) |  | 0–1 (n=2) |  |
| Lee et al. (2008) | 2 | 8 | Etest, BMD | TSB | 35⁰ | 40 | 0–1 (n=2) |  | 0–1 (n=2) |  |
| Lee et al. (2023) | 1 | 1 | VITEK | CAMHB | 37⁰ | 7 | 0.25 (n=1) |  | 0.25 (n=1) |  |
| Lenhard et al. (2016) | 2 | 32 | NA | CAMHB | 37⁰ | 192 | 0–6 (n=9) |  | 0–3 (n=2) |  |
| Lenhard et al. (2017) | 3 | 52 | CLSI | MHB | 37⁰ | 314 | 0–125 (n=15) | 8g over 3h q8h; 2g over 3h q8h; 4g over 3h q8h; 6g over 3h q8h | 0–3.1 (n=2) | 2.22mg/kg and 1.43mg/kg q12h; 1.43mg/kg q12h |
| Lenhard et al. (2017) | 2 | 8 | NA | CAMHB | NA | 48 | 0–0.86 (n=2) |  | 0–0.05 (n=3) |  |
| Lenhard et al. (2017) | 1 | 4 | CLSI | CAMHB | NA | 8 | 0 (n=1) | 2g over 3h q8h | 0 (n=1) | 3.33mg/kg and 1.43mg/kg q12h |
| Li et al. (2020) | 6 | 36 | Agar dilution | CAMHB | 37⁰ | 180 | 0–1 (n=3) |  | 0–1 (n=4) |  |
| Liang et al. (2011) | 2 | 12 | BMD | CAMHB | 35⁰ | 96 | 0–0.5 (n=4) |  | 0–0.5 (n=2) |  |
| Lim et al. (2009) | 2 | 8 | BMD | CAMHB | 35⁰ | 48 | 0–2 (n=3) |  | 0–0.5 (n=2) |  |
| Luna-De-Alba et al. (2024) | 1 | 4 | Disk diffusion, BMD | MHB | 37⁰ | 20 | 0–0.25 (n=3) |  | 0–0.25 (n=3) |  |
| Oliva et al. (2019) | 2 | 10 | VITEK | CAMHB | 37⁰ | 60 | 0–1 (n=3) |  | 0–1 (n=2) |  |
| Smith et al. (2020) | 1 | 8 | BMD | CAMHB | NA | 56 | 0 (n=1) | 4g over 3h q8h | 0 (n=1) | 2.22mg/kg and 1.43mg/kg q12h; 1.11mg/kg and 0.72mg/kg q12h; 3.71mg/kg and 2.40mg/kg q12h; 7.43mg/kg and 4.80mg/kg q12h; 14.8mg/kg and 9.60mg/kg q12h |
| Tängdén et al. (2017) | 4 | 16 | Etest | CAMHB | NA | 112 | 0 (n=1) | 1g | 0 (n=1) | 2.22 mg/kg |
| Wences et al. (2022) | 4 | 52 | CLSI | CAMHB | 37⁰ | 364 | 0–4 (n=6) |  | 0–4 (n=6) |  |
| Wong et al. (2020) | 3 | 12 | BMD | CAMHB | 35⁰ | 50 | 0–0.62 (n=2) |  | 0–4 (n=3) |  |
| Yang et al. (2016) | 4 | 16 | BMD | MHB | 37⁰ | 48 | 0–1 (n=5) |  | 0–1 (n=2) |  |

BMD – broth microdilution; CAMHB – cation-adjusted Mueller-Hinton broth; CLSI – MIC measurement according to CLSI guidelines; MHB – Mueller-Hinton broth; MIC – minimum inhibitory concentration in mg/L; NA – not available; TSB – tryptic soy broth.

**Abdul-Mutakabbir JC, Yim J, Nguyen L, Maassen PT, Stamper K, Shiekh Z, Kebriaei R, Shields RK, Castanheira M, Kaye KS, Rybak MJ.** In vitro synergy of colistin in combination with meropenem or tigecycline against carbapenem-resistant Acinetobacter baumannii. Antibiotics (Basel). 2021;10(7):880. doi:10.3390/antibiotics10070880.

**Abdul-Mutakabbir JC, Sakyi Opoku N, Tan KK, Jorth P, Nizet V, Fletcher HM, Kaye KS, Rybak MJ.** Determining susceptibility and potential mediators of resistance for the novel polymyxin derivative, SPR206, in Acinetobacter baumannii. Antibiotics (Basel). 2024 Jan 4;13(1):47. doi:10.3390/antibiotics13010047.

**Bedenić B, Beader N, Godič-Torkar K, Prahin E, Mihaljević L, Ćačić M, Vraneš J.** Postantibiotic effect of colistin alone and combined with vancomycin or meropenem against Acinetobacter spp. with well defined resistance mechanisms. J Chemother. 2016 Oct;28(5):375-382. doi:10.1179/1973947815Y.0000000062.

**Bian X, Liu X, Chen Y, Chen D, Li J, Zhang J.** Dose Optimization of Colistin Combinations against Carbapenem-Resistant Acinetobacter baumannii from Patients with Hospital-Acquired Pneumonia in China by Using an In Vitro Pharmacokinetic/Pharmacodynamic Model. Antimicrobial Agents and Chemotherapy. 2019;63(4):e01989-18. doi:10.1128/AAC.01989-18.

**Ju YG, Lee HJ, Yim HS, Lee MG, Sohn JW, Yoon YK.** In vitro synergistic antimicrobial activity of a combination of meropenem, colistin, tigecycline, rifampin, and ceftolozane/tazobactam against carbapenem-resistant Acinetobacter baumannii. Sci Rep. 2022 May 9;12(1):7541. doi:10.1038/s41598-022-11464-6.

**Lee CH, Tang YF, Su LH, Chien CC, Liu JW.** Antimicrobial effects of varied combinations of meropenem, sulbactam, and colistin on a multidrug-resistant Acinetobacter baumannii isolate that caused meningitis and bacteremia. Microb Drug Resist. 2008 Sep;14(3):233-237. doi:10.1089/mdr.2008.0840.

**Lee JH, Kim J, Kim G-Y.** Synergistic effects of a probiotic culture extract and antimicrobial combinations against multidrug-resistant Acinetobacter baumannii. Medicina (Kaunas). 2023 May 15;59(5):947. doi:10.3390/medicina59050947.

**Lenhard JR, Gall JS, Bulitta JB, Thamlikitkul V, Landersdorfer CB, Forrest A, Nation RL, Li J, Tsuji BT.** Comparative pharmacodynamics of four different carbapenems in combination with polymyxin B against carbapenem-resistant Acinetobacter baumannii. Int J Antimicrob Agents. 2016 Dec;48(6):719-724. doi:10.1016/j.ijantimicag.2016.07.024.

**Lenhard JR, Bulitta JB, Connell TD, King-Lyons N, Landersdorfer CB, Cheah SE, Thamlikitkul V, Shin BS, Rao G, Holden PN, Walsh TJ, Forrest A, Nation RL, Li J, Tsuji BT.** High-intensity meropenem combinations with polymyxin B: new strategies to overcome carbapenem resistance in Acinetobacter baumannii. J Antimicrob Chemother. 2017 Jan;72(1):153-165. doi:10.1093/jac/dkw355.

**Lenhard JR, Smith NM, Bulman ZP, Tao X, Thamlikitkul V, Shin BS, Nation RL, Li J, Bulitta JB, Tsuji BT.** High-dose ampicillin-sulbactam combinations combat polymyxin-resistant Acinetobacter baumannii in a hollow-fiber infection model. Antimicrob Agents Chemother. 2017 Mar;61(3):e01268-16. doi:10.1128/AAC.01268-16.

**Lenhard JR, Thamlikitkul V, Silveira FP, Garonzik SM, Tao X, Forrest A, Shin BS, Kaye KS, Bulitta JB, Nation RL, Li J, Tsuji BT.** Polymyxin-resistant, carbapenem-resistant Acinetobacter baumannii is eradicated by a triple combination of agents that lack individual activity. Journal of Antimicrobial Chemotherapy. 2017;72(5):1415–1420. doi:10.1093/jac/dkx002.

**Li J, Fu Y, Zhang J, Zhao Y, Fan X, Yu L, Wang Y, Zhang X, Li C.** The efficacy of colistin monotherapy versus combination therapy with other antimicrobials against carbapenem-resistant Acinetobacter baumannii ST2 isolates. J Chemother. 2020 Nov;32(7):359-367. doi:10.1080/1120009X.2020.1764282.

**Liang W, Liu XF, Huang J, Zhu DM, Li J, Zhang J.** Activities of colistin- and minocycline-based combinations against extensive drug resistant Acinetobacter baumannii isolates from intensive care unit patients. BMC Infect Dis. 2011 Apr 27;11:109. doi:10.1186/1471-2334-11-109.

**Lim TP, Tan TY, Lee W, Sasikala S, Tan TT, Hsu LY, Kwa AL.** In vitro activity of various combinations of antimicrobials against carbapenem-resistant Acinetobacter species in Singapore. J Antibiot (Tokyo). 2009 Dec;62(12):675-679. doi:10.1038/ja.2009.99.

**Luna-De-Alba A, Flores-Treviño S, Camacho-Ortiz A, Contreras-Cordero JF, Bocanegra-Ibarias P.** Genetic characterization of multidrug-resistant Acinetobacter baumannii and synergy assessment of antimicrobial combinations. Antibiotics (Basel). 2024 Nov 13;13(11):1079. doi:10.3390/antibiotics13111079.

**Oliva A, Garzoli S, De Angelis M, Marzuillo C, Vullo V, Mastroianni CM, Ragno R.** In-vitro evaluation of different antimicrobial combinations with and without colistin against carbapenem-resistant Acinetobacter baumannii. Molecules. 2019 Mar 3;24(5):886. doi:10.3390/molecules24050886.

**Smith NM, Lenhard JR, Boissonneault KR, Landersdorfer CB, Bulitta JB, Holden PN, Forrest A, Nation RL, Li J, Tsuji BT.** Using machine learning to optimize antibiotic combinations: dosing strategies for meropenem and polymyxin B against carbapenem-resistant Acinetobacter baumannii. Clin Microbiol Infect. 2020 Sep;26(9):1207-1213. doi:10.1016/j.cmi.2020.02.004.

**Tängdén T, Karvanen M, Friberg LE, Odenholt I, Cars O.** Assessment of early combination effects of colistin and meropenem against Pseudomonas aeruginosa and Acinetobacter baumannii in dynamic time-kill experiments. Infect Dis (Lond). 2017 Jul;49(7):521-527. doi:10.1080/23744235.2017.1296183.

**Wences M, Wolf ER, Li C, Singh N, Bah N, Tan X, Huang Y, Bulman ZP.** Combatting planktonic and biofilm populations of carbapenem-resistant Acinetobacter baumannii with polymyxin-based combinations. Antibiotics (Basel). 2022 Jul 16;11(7):959.

**Wong FHS, Cai Y, Leck H, Lim TP, Teo JQM, Lee W, Koh TH, Tan TT, Tan KW, Kwa AL-H.** Determining the development of persisters in extensively drug-resistant Acinetobacter baumannii upon exposure to polymyxin B-based antibiotic combinations using flow cytometry. Antimicrob Agents Chemother. 2020 Feb 21;64(3):e01712-19. doi:10.1128/AAC.01712-19.

**Yang Y-S, Lee Y, Tseng K-C, Huang W-C, Chuang M-F, Kuo S-C, Yang Lauderdale T-L, Chen T-L.** In vivo and in vitro efficacy of minocycline-based combination therapy for minocycline-resistant Acinetobacter baumannii. Antimicrob Agents Chemother. 2016 Jul;60(7):4047-4054. doi:10.1128/AAC.02994-15.

**Table S2**. Modelling steps and each steps best-fitting model shown using asterisk

|  | **Description** | **OFV** |
| --- | --- | --- |
| **Growth control models** | | |
| G1* | IIV only on carrying capacity | -33.55 |
| G2 | IIV only on inoculum | 115.93 |
| G3 | IIV only on growth rate | 509.50 |
| G4 | IIV only on carrying capacity, experiment type as a covariate for growth rate | terminated |
| G5 | IIV only on carrying capacity, experiment type as a covariate for carrying capacity | -36.47 |
| **Colistin/polymyxin B model** | | |
| P1 | No covariates, IIV on EC50 | 1029.70 |
| P2 | No covariates, IIV on Emax | 1207.02 |
| P3 | Inoculum effect (continuous), IIV on EC50 | 1024.93 |
| P4 | Inoculum effect (only for 10^8^), IIV on EC50 | 1005.534 |
| P5 | Inoculum effect (only for 10^8^), power term of EC50=f(MIC) estimated, IIV on EC50 | 1005.57 |
| P6* | Inoculum effect (only for 10^8^), linear term of EC50=f(MIC) estimated, IIV on EC50 | 985.40 |
| P7 | Inoculum effect (only for 10^8^), linear term of EC50=f(MIC) estimated separately for colistin and polymyxin B, IIV on EC50 | 984.33 |
| P8 | Inoculum effect (only for 10^8^), linear term of EC50=f(MIC) estimated, IIV on EC50, experiment type as a covariate for attenuation rate | terminated |
| **Meropenem model** | | |
| M1 | No covariates, IIV on EC50 | 436.09 |
| M2 | No covariates, IIV on Emax | 512.52 |
| M3 | Inoculum effect (continuous), IIV on EC50 | 380.50 |
| M4 | Inoculum effect (continuous), power term of EC50=f(MIC) estimated, IIV on EC50 | 380.30 |
| M5 | Inoculum effect (continuous), linear term of EC50=f(MIC) estimated, IIV on EC50 | 379.85 |
| M6* | Inoculum effect (continuous), IIV on EC50, acquired beta-lactamase as a covariate for Emax | 361.92 |
| M7 | Inoculum effect (continuous), IIV on EC50, acquired beta-lactamase as a covariate for EC50 | 379.54 |
| M8 | Inoculum effect (continuous), IIV on EC50, acquired beta-lactamase as a covariate for Emax, experiment type as a covariate for attenuation rate | large gradients |
| **Combination model** | | |
| C1 | No general pharmacodynamic interaction | 3780.84 |
| C2 | Colistin/polymyxin B reduces meropenem EC50 | 3681.97 |
| C3 | Colistin/polymyxin B increases meropenem Emax | 3647.45 |
| C4 | Colistin/polymyxin B reduces meropenem EC50, IIV on the extent | terminated |
| C5 | Colistin/polymyxin B increases meropenem Emax, IIV on the extent | terminated |
| C6* | Colistin/polymyxin B reduces meropenem EC50 (extent fixed to 1) and increases meropenem Emax | 3622.26 |
| C7 | Colistin/polymyxin B reduces meropenem EC50 (extent estimated) and increases meropenem Emax | 3621.33 |
| C8 | Colistin/polymyxin B reduces meropenem EC50 (extent fixed to 1) and increases meropenem Emax, acquired beta-lactamase modifies colistin/polymyxin B effect on meropenem Emax | 3622.25 |
| C9 | Colistin/polymyxin B reduces meropenem EC50 (extent fixed to 1) and increases meropenem Emax, acquired beta-lactamase modifies colistin/polymyxin B effect on meropenem EC50 (extent estimated) | 3607.78, RSE>300% |

Experiment type refers to static or dynamic setting. IIV – interisolate variability; OFV – objective function value; RSE – relative standard error.


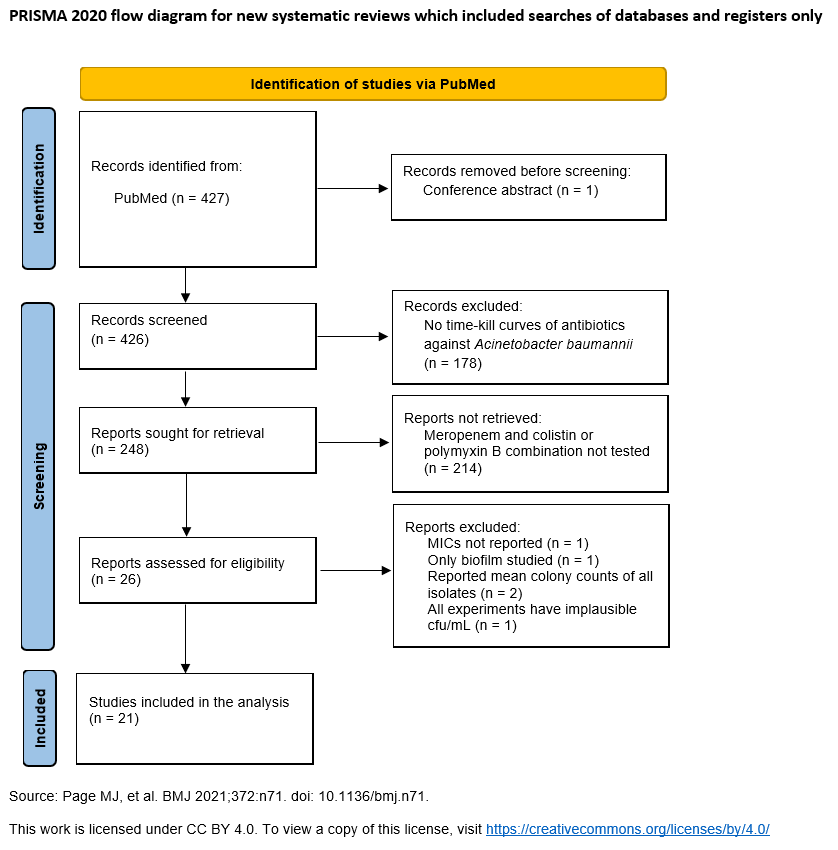


**Figure S1**. PRISMA flowchart of including studies in the analysis.
cfu – colony forming unit; MIC – minimum inhibitory concentration.


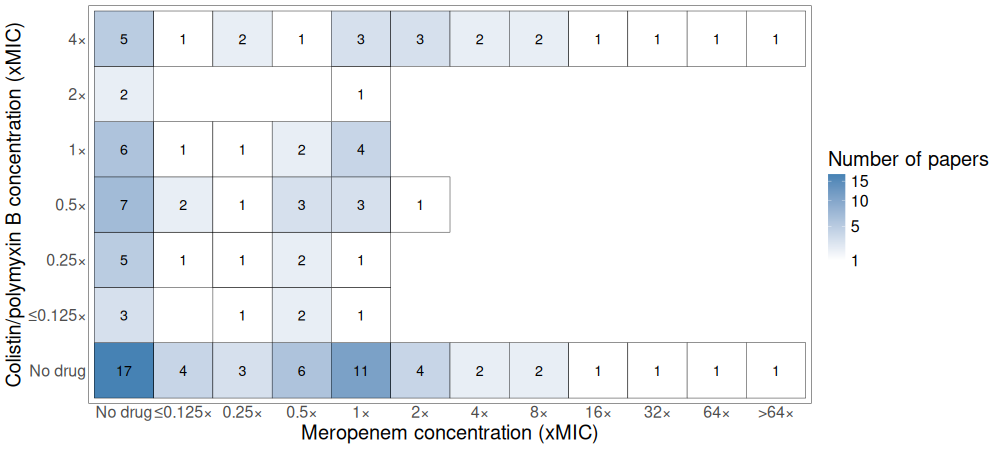
**Figure S2**. Meropenem and colistin/polymyxin B concentrations tested in static time-kill experiments, expressed as multiples of isolate-specific MIC. Numbers of papers using this MIC combination are shown for each concentration combination.


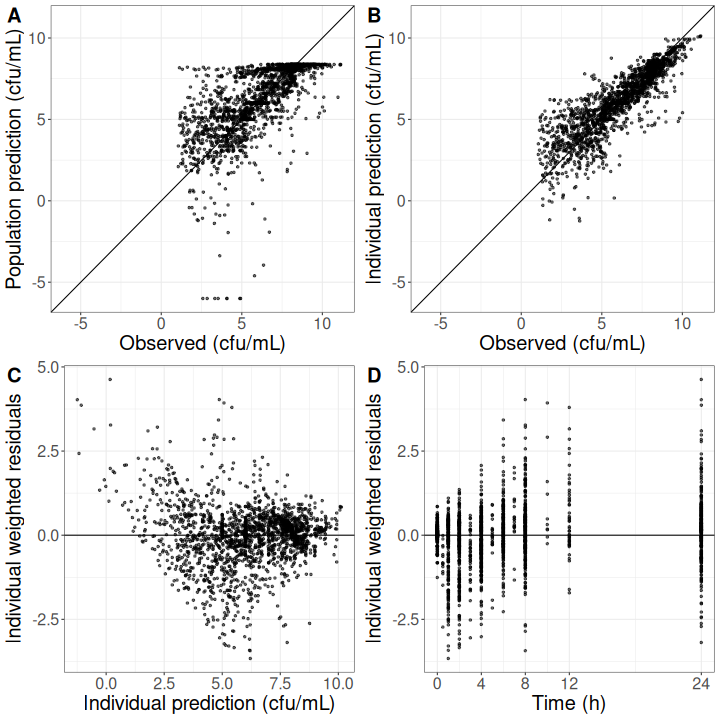


**Figure S3**. Goodness-of-fit plots of the final pharmacokinetic-pharmacodynamic model. A) Observed vs population predictions. B) Observed vs individual predictions. C) Individual weighted residuals vs individual predictions, D) Individual weighted residuals vs time after the start of experiment. Observations below limit of quantification are excluded from the plots.


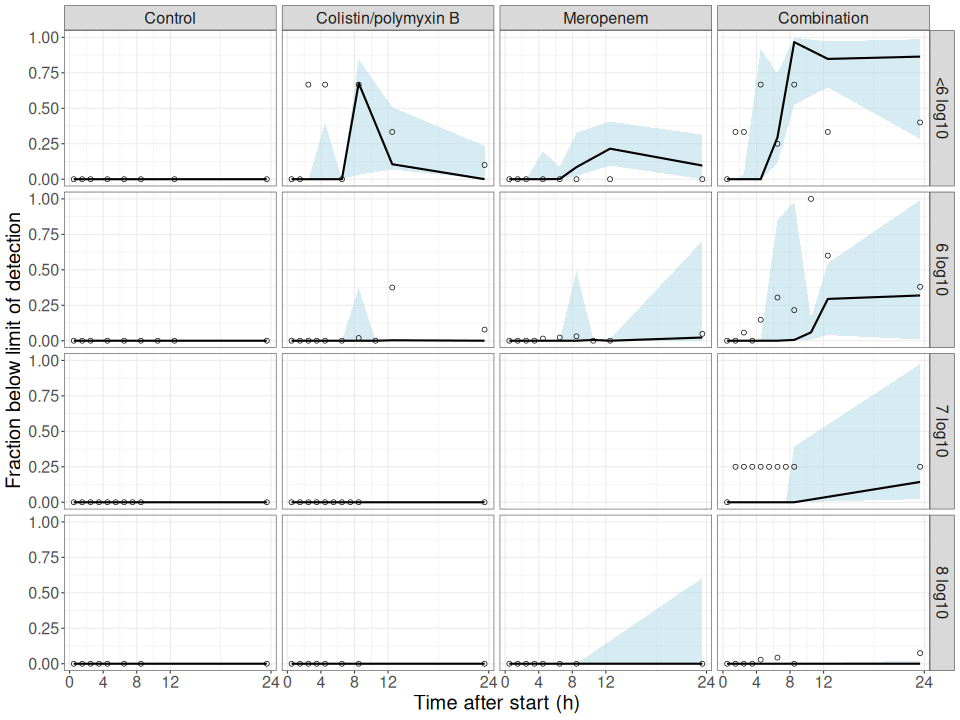


**Figure S4**. Posterior predictive check of fraction of observations below limit of quantification (BLOQ). Observed fraction of BLOQ measurements, shown in dots, is compared with the simulated distribution of BLOQ fractions across time (median shown by solid line, 90% prediction interval from 5^th^ to 95^th^ percentile by shaded area), stratified by treatment group and inoculum where applicable.


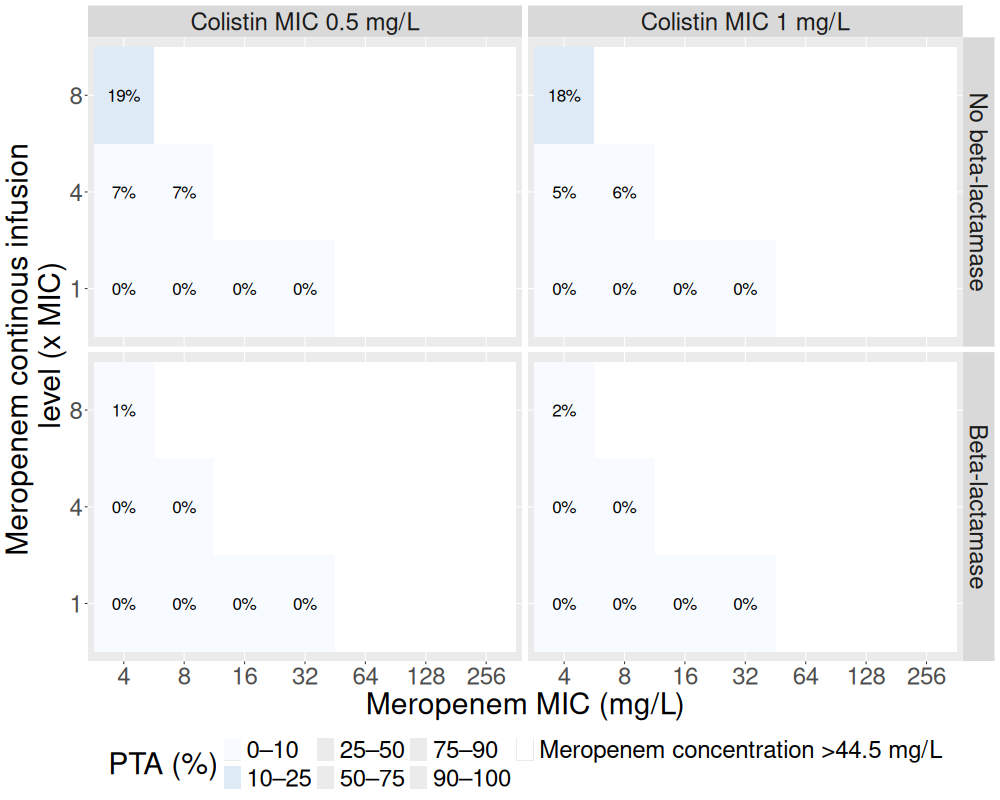


**Figure S5**. Probability of target (defined as bacterial count below the limit of quantification of 10 colony-forming units/mL) attainment (PTA) for meropenem administered by continuous infusion achieving different levels of multiples of meropenem minimum inhibitory concentration (MIC) as monotherapy, stratified by meropenem MIC, colistin MIC, and the presence of acquired beta-lactamase. Tiles indicate whether PTA ≥0.9 was achieved at 24 h for each steady-state meropenem concentration expressed as a multiple of the MIC. Tile colour represents the extent of PTA (darker colours indicate higher PTA) and percentage shows PTA value. White tiles denote exposure combinations exceeding the predefined meropenem steady-state toxicity threshold (>44.5 mg/L) and were therefore considered infeasible.


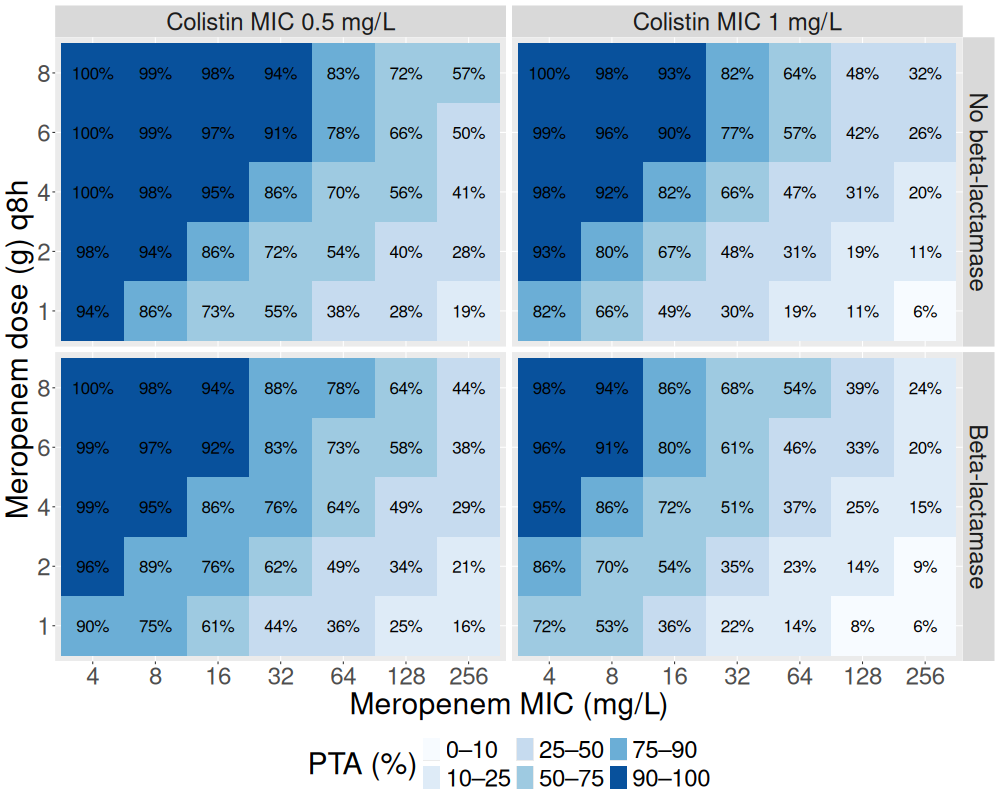


**Figure S6**. Probability of target (defined as bacterial count at 24 h ≥2 log10 lower than inoculum) attainment (PTA) for meropenem administered as intermittent dosing meropenem, stratified by meropenem minimum inhibitory concentration (MIC), colistin MIC, and the presence of acquired beta-lactamase. Tiles indicate whether PTA ≥0.9 was achieved at 24 h for each meropenem dosing regimen. Tile colour represents the extent of PTA (darker colours indicate higher PTA) and percentage shows PTA value.


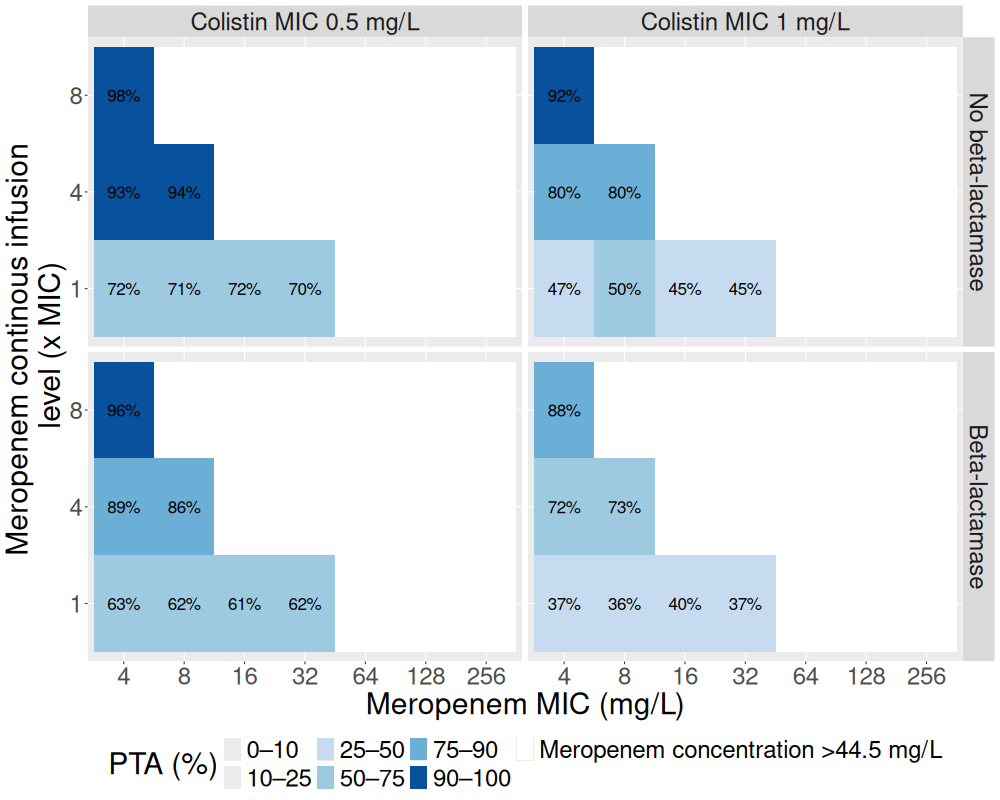


**Figure S7**. Probability of target (defined as bacterial count at 24 h ≥2 log10 lower than inoculum) attainment (PTA) for meropenem administered by continuous infusion achieving different levels of multiples of meropenem minimum inhibitory concentration (MIC) as monotherapy, stratified by meropenem MIC, colistin MIC, and the presence of acquired beta-lactamase. Tiles indicate whether PTA ≥0.9 was achieved at 24 h for each steady-state meropenem concentration expressed as a multiple of the MIC. Tile colour represents the extent of PTA (darker colours indicate higher PTA) and percentage shows PTA value. White tiles denote exposure combinations exceeding the predefined meropenem steady-state toxicity threshold (>44.5 mg/L) and were therefore considered infeasible.
